# Holistic Management of Adult ADHD with a History of Addiction: Emphasis on Low-Addiction-Risk Psychopharmacotherapy

**DOI:** 10.3390/jcm14186470

**Published:** 2025-09-14

**Authors:** Kacper Żełabowski, Wiktor Petrov, Dawid Ślebioda, Malwina Rusinek, Kamil Biedka, Katarzyna Błaszczyk, Michał Wesołowski, Kacper Wojtysiak, Mateusz Sroka, Zuzanna Ratka, Ignacy Ilski, Agnieszka Chłopaś-Konowałek

**Affiliations:** 1 Scientific Society for Psychopharmacology, Department of Forensic Medicine, Wroclaw Medical University, 4 J. Mikulicza-Radeckiego Street, 50345 Wroclaw, Poland; kacper.zelabowski@outlook.com (K.Ż.); wiktor.petrov@student.umw.edu.pl (W.P.); dawid.slebioda@student.umw.edu.pl (D.Ś.); malwina.rusinek@student.umw.edu.pl (M.R.); kacper.wojtysiak@student.umw.edu.pl (K.W.); mateusz.sroka@student.umw.edu.pl (M.S.); zuzanna.ratka@student.umw.edu.pl (Z.R.); ignacy.ilski@student.umw.edu.pl (I.I.); 2 Department of Physiology and Pathophysiology, Division of Pathophysiology, Wroclaw Medical University, Chalubinskiego 10, 50368 Wroclaw, Poland; kamil.biedka@umw.edu.pl (K.B.); michal.wesolowski@umw.edu.pl (M.W.); 3 Department of Physiology and Pathophysiology, Division of Physiology, Wroclaw Medical University, Chalubinskiego 10, 50368 Wroclaw, Poland; blaszczykrzyk@gmail.com; 4 Department of Forensic Medicine, Division of Molecular Techniques, Wroclaw Medical University, Sklodowskiej-Curie 52, 50369 Wroclaw, Poland

**Keywords:** ADHD, adult, substance use disorder, psychopharmacotherapy, stimulants, non-stimulants, pharmacological treatment, non-pharmacological treatment

## Abstract

Attention-deficit/hyperactivity disorder (ADHD) is a neurodevelopmental disorder generally associated with pediatric patients and in lesser extent with adults. Patients diagnosed with ADHD have a higher likelihood of developing addiction. Consequently, a disorder that frequently co-occurs with ADHD is Substance Use Disorder (SUD). The pharmaceuticals prescribed in the treatment of ADHD are predominantly stimulants, such as methylphenidate and amphetamines, which possess a high addiction potential. The objective of this study is to examine the risk of developing substance dependence during stimulant treatment in individuals with ADHD who have a natural predisposition to addiction, with particular emphasis on adult patients with a history of SUD. Our literature review was conducted using research papers from PubMed, Google Scholar, Embase, ProQuest and ScienceDirect. The main results from our review are as follows: (i) the majority of studies indicate that the administration of stimulants in the treatment of ADHD does not increase the risk of developing Substance Use Disorder; (ii) stimulants may also be used in the treatment of SUD; (iii) while pharmacotherapy is a crucial part of ADHD treatment, a holistic approach comprising pharmacological and non-pharmacological therapy is most effective; (iv) holistic management of ADHD is necessary to improve patients’ quality of life to the greatest extent possible.

## 1. Introduction

Attention-deficit/hyperactivity disorder (ADHD) is a prevalent neurodevelopmental disorder. The disorder is predominantly observed in the pediatric population. The prevalence of the condition among children and adolescents worldwide is estimated to range from 6 to 10% of the global population [1]. In different parts of the world that number can vary. For example, in Poland, the ADHD population is estimated to range from 3 to 12% [2]. In the United States this number is approximately 6% [3]. As for Africa, current estimations put the prevalence of ADHD in the general population at 1.5% [4].

The mean time of onset of initial symptoms varies between the ages of 2 and 8, with a final diagnosis occurring on average between 6 and 18 years of age [5]. Between 60% and 70% of adults diagnosed with ADHD in childhood continue to experience symptoms in adulthood [6]. ADHD is a condition that, if left untreated or improperly diagnosed, can have a detrimental impact on numerous aspects of an individual’s life, adversely affecting their overall quality of life. The condition has been shown to manifest in various domains, leading to challenges in multiple areas of life, including academic, social and familial spheres. Adult patients diagnosed with this condition are more likely to be divorced, unemployed and they are more prone to being involved in traffic accidents [7,8].

According to the guidelines established by the National Institute for Health and Care Excellence (NICE), the treatment of ADHD should commence with the administration of amphetamine derivatives, which exert their effects on the central nervous system (CNS) through the dopaminergic pathway. These interventions have been demonstrated to be remarkably efficacious in the alleviation of ADHD symptoms [9]. In Poland, the pharmaceutical agent of primary choice for the treatment of ADHD is methylphenidate, a stimulant with a high addiction potential [10]. Substance Use Disorder (SUD) involving psychoactive substances is the most commonly reported comorbid condition in individuals with ADHD [11]. This suggests the necessity to limit the administration of stimulant medications used to treat the specified disorder, and alternatively, to increase the utilization of alternative pharmaceutical agents that possess a reduced potential for inducing addiction. Second-line drugs are CNS non-stimulants including propylamine derivatives such as atomoxetine. They are primarily recommended for patients suffering from mood disorders, anxiety disorders and a tendency towards addiction [10].

The aim of this paper is to present treatment strategies for ADHD in adult patients with a history of substance dependence, with a presentation of pharmacotherapy including stimulants, especially modified forms that maintain clinical effectiveness but carry a lower risk of developing addiction, such as methylphenidate transdermal patches [12], and drugs with low addiction potential, which include, among others, atomoxetine and bupropion.

## 2. ADHD Characteristics in Adults

### 2.1. Epidemiology of Adult ADHD

The prevalence of attention-deficit/hyperactivity disorder among adults varies between 2% and 6% on a global scale [6]. In childhood and adolescence, the ratio of diagnosed cases of ADHD in male subjects to female subjects is at a minimum of 3:1. In contrast, among adults, Salvi et al. presented the male-to-female ratio as being approximately 2:1 [13], while da Silva et al. estimated it to be 1:1 [14]. The discrepancy in data between the pediatric and adult populations can be attributed to the underdiagnosis of ADHD in girls, given the prevalence of the condition in the female demographic. This phenomenon can be attributed to the observation that, in boys, the most prevalent symptoms—namely, motor hyperactivity and impulsivity—are more pronounced, while in girls, the less noticeable attention deficit is predominant [13,15,16].

### 2.2. Symptoms of ADHD in Adulthood

The hallmark triad of ADHD symptoms comprises the following: (i) hyperactivity; (ii) impulsivity; and (iii) inattention. In adulthood, the easily observable hyperactivity tends to diminish, whereas impulsivity and difficulty focusing attention predominate [17]. This underscores the complexity of diagnosing ADHD in adults, which can differ significantly from the diagnosis of ADHD in children. A higher prevalence of concomitant psychiatric disorders has been observed in adults, with the potential for such disorders to obscure the manifestations of ADHD. These include anxiety, emotional, mood, and personality disorders. Subjects in this group also exhibit sleep disturbances and a proclivity towards abusing addictive substances, including but not limited to alcohol, cocaine, and opioids [6,18,19].

### 2.3. Adult ADHD Diagnosis

The most recent diagnosis of ADHD is based primarily on the International Classification of Diseases, 11th edition (ICD-11) and the Diagnostic and Statistical Manual of Mental Disorders, 5th edition (DSM-5), which is the classification system dedicated exclusively to mental disorders. Nonetheless, the diagnostic criteria delineated in the DSM-V have been the subject of substantial criticism, particularly with regard to disorders that manifest in children [20]. The 11th edition of the ICD is relatively new—it was published in 2018 [21]. According to the criteria outlined in the ICD-11 and DSM-V guidelines, a diagnosis of ADHD is made when characteristic symptoms manifest by the age of 12. These symptoms include hyperactivity, impulsivity, and inattention. The implementation of these criteria serves to further complicate the diagnosis of this condition in adulthood [7].

Diagnostic criteria of ADHD according to ICD-11 and DSM-5 are concluded in Table 1.

### 2.4. Consequences of Untreated ADHD

Untreated ADHD has been shown to result in the occurrence of various functional disorders that affect numerous spheres of life. Due to inattention, problems with the effective performance of planned tasks were observed, as well as a decrease in efficiency while studying. These challenges can further reduce patients’ ability to work and cause a higher rate of unemployment among adults with ADHD [22]. A study conducted in 2003 examined the relationship between attention-deficit/hyperactivity disorder and income, focusing on a sample of 500 individuals with ADHD and 501 without ADHD. The study revealed that individuals with ADHD in the United States typically have annual incomes that are approximately $10,000 less than individuals without ADHD [23]. As mentioned above, individuals diagnosed with ADHD are more likely to face unemployment compared to those without such a diagnosis. The underlying cause of this phenomenon, apart from inattention, may be attributed to impaired motor coordination, as well as difficulties with verbal fluency and executive planning [24]. Attention-deficit/hyperactivity has a considerable impact on interpersonal relationships, as evidenced by data on divorce rates. Biederman et al. revealed in their study that individuals with ADHD exhibited a significantly higher rate of divorce compared to the control group, with a 28% divorce rate among those with ADHD compared to 15% among the control group without ADHD [25]. A rise in criminal activity has been observed among both adolescent and adult patients which may be driven by their increased impulsivity [26]. It is estimated that up to 25% of offenders in prison, police custody, under probation supervision, or under court-ordered psychiatric care have ADHD [27]. Dysfunction occurs primarily when the patient makes his or her own decisions about plans and goals. Properly selected treatment helps to minimize not only the classic symptoms of ADHD, but also such difficulties [22]. In the treatment of ADHD, psychopharmacotherapy is the most significant component, while non-pharmacological methods, when used alongside pharmacotherapy, improve patient’s quality of life. Among the most efficacious of these are cognitive behavioral therapy (CBT), mindfulness-based interventions (MBI), and cognitive treatments [28,29].

### 2.5. The History of the Term “ADHD”

At the turn of the 1900s, reports of children with attentional and behavioral issues more easily suggested “defective moral control” than neurobiological processes. At such periods of time in the USA and Europe, educational and medical discourse did label children of this sort as “troublesome” but did not label such behavior as psychiatric. Therapeutic strategies at the time were largely founded on disciplinary or pedagogical interventions, including structured environments, moral instruction, and the administration of sedatives or tonics. In 1980, under DSM-III, the term “attention-deficit disorder (ADD) with/without hyperactivity” was used, and then in 1987 the term “attention-deficit/hyperactivity disorder” appeared for the first time in DSM-III-R [30]. The clinical descriptions of a syndrome, similar to ADHD, were provided by George F. Still in his Goulstonian lectures that appeared in The Lancet in 1902 [31]. The first use of stimulants (Benzedrine/amphetamine sulfate) in children with disturbance of behavior was described by Charles Bradley in 1937 in the USA [30]. In European classification, names have traditionally been distinct from the American system (ICD-10: “hyperkinetic disorders”) based on the European diagnostic focus, e.g., in Poland. Harmony was brought about by ICD-11 [21]. It should be mentioned that in the early 20th century, in each of the three countries, management was basically pedagogical, behavioral, and somatic (hygiene, discipline, general sedatives), while current stimulant pharmacotherapy started at the earliest in the USA (Bradley), and later in the UK and Poland, depending on the availability of preparations and the development of diagnostic paradigms [21,30].

## 3. The Impact of Personality Traits and ADHD Symptoms on the Frequency of Addiction

Impulsive behavior (also referred to as “disinhibition”) and sensation-seeking (risk-taking) are two components of impulsivity that have been associated with ADHD and SUD. The aforementioned psychological constructs are mediated by inhibitory brain networks for the purpose of processing motivation and reward. Impulsivity is defined as “reflexive and inappropriate behavior referring to the inability to inhibit or suppress a strong impulse despite the intention to stop”. It is characterized by rapid, unplanned reactions to stimuli with little anticipation of consequences [32]. It defines a mechanism in which executive control and motivational-affective deficits that are a component of ADHD may contribute to increased susceptibility to SUD [33]. Research conducted on both human and animal subjects has demonstrated that the regions of the brain responsible for regulating impulsive behavior coincide with those that are critical for the initiation of SUDs. In the context of addiction, impulsivity is theorized to function as a risk factor for the initiation of substance abuse, as well as a factor associated with the propensity for relapse [34]. A growing body of research has identified the frontal-striatal and frontoparietal regions as key components of the inhibitory control network. This finding is supported by functional magnetic resonance imaging (fMRI) studies that incorporate “inhibitory control tasks” within their methodology [35]. Increased disinhibition and abnormalities in the aforementioned brain regions were identified in patients with ADHD and SUD. Abnormalities in the networks between the ventral striatum, anterior cingulate cortex, orbitofrontal cortex, and ventral medial prefrontal cortex appear to be involved in the development of both disorders [36]. Furthermore, research on impulsivity has demonstrated hypoactivity during inhibitory control of impulsive behavior, reduced coherence of white matter microstructure [37] and reduced cortical thickness of the caudal inferior frontal gyrus in individuals with ADHD [38]. Dopaminergic activity in the reward network underlies the desire to seek intense sensations [39]. In addition to an increased propensity for disinhibition, it has also been found that individuals with SUDs and adults with ADHD show an increased tendency toward large sensations [32,40]. The findings, when considered collectively, demonstrate that high impulsivity is the predominant component shared in the concurrent development of ADHD and SUD. In addition to an increased propensity for disinhibition, it has also been found that individuals with SUDs and adults with ADHD show an increased tendency toward large sensations [40]. The findings, when considered collectively, show that high impulsivity is the predominant component shared in the concurrent development of ADHD and SUD.

Impulsivity is a multifaceted construct, comprising several distinct traits. A study suggested that affect-based and non-planning impulsivity are associated with increased risk for substance dependence, whereas choice and motor impulsivity are not consistently associated with this disorder [41].

It is noteworthy that, in addition to impulsivity, the role of compulsivity has been recognized. Compulsivity can be defined as repetitive actions, often driven by a top-down sense of wanting to perform an activity [42], which is “inappropriate to the situation and has no obvious connection to the overall goal” [43]. This phenomenon is significant in the later stages of the disorder, as the sufferer’s behavior is driven by a desire to avoid negative experiences [44]. Although ADHD is not generally classified as a compulsive disorder, it is recognized that it may fall within the spectrum of obsessive-compulsive disorders [45].

Individuals diagnosed with ADHD exhibit a higher prevalence of cigarette smoking, alcohol consumption, and psychostimulant use compared to individuals without ADHD [46,47].

Chronic drug exposure to such drugs as nicotine, alcohol, and psychostimulants has profound adaptive actions on the brain reward system. It leads to downregulation of striatal dopamine D_2_ receptors and inadequate signaling. These adaptations contribute centrally to the development of long-term addiction and heightened impulsivity [48]. Nicotine, specifically, leads to desensitization of nicotinic acetylcholine receptors (nAChRs), and thus compensatory upregulation, dopaminergic release potentiation and vicious cycle of addiction [49,50]. Alcohol interferes with the balance between glutamatergic excitation and GABAergic inhibition, disrupting dopaminergic neuron firing and reward processing [51].

Specifically, the initiation of cigarette smoking in adolescence has been well established to be an important predictor of alcohol and drug dependence development [52]. Patients with ADHD thus may have a greater risk of developing addictions. In males, ADHD has also been reported as a significant predictor of premature initiation of cigarette smoking (age less than 15 years) and heightened risk of nicotine consumption [46]. Furthermore, patients with ADHD comorbid behavioral disorders like anxiety or mood instability accounted for the bulk of smokers [53]. A study demonstrated that children exposed to prenatal maternal tobacco smoking are 71% more likely to suffer from ADHD. Nicotine can affect fetal brain development directly, through the dopaminergic pathway that is closely associated with ADHD symptoms, or indirectly, by causing hypoxia and a chronic inflammatory response [54].

When it comes to antidepressants, other research showed in their systematic review that there is no conclusive evidence maternal antidepressant usage during pregnancy increases the offspring’s risk of ADHD [55].

In the neurophysiological regard, comorbidity of anxiety and mood instability in ADHD is associated with dysfunction in neurotransmitter systems. ADHD kids tend to have more glutamate and less GABA, which lead to cortical hyperexcitability and aberrant inhibitory control [56]. GABA deficiency has been found to contribute to heightened arousal and inadequate regulation of varied emotional responses, whereas glutamatergic hyperfunction was found to be the basis of stress reactivity and anxiety. Hyperactivation of the noradrenergic system was found to be connected with heightened sensitivity to stress, which in its turn strengthens anxiety symptoms [57]. PET scans have also demonstrated the reduction in norepinephrine transporter (NET) availability in attention- and emotion-processing brain area neurons. That is associated with compromised mood and arousal regulation [58].

In brief, chronic nicotine, alcohol and other stimulant use has been found to bring about alterations in the brain that create a diminished perception of natural rewards together with an escalation of impulsive behaviors. Brain chemistry is altered through the depletion of these vital receptors from prolonged exposure to alcohol and nicotine. This has been found to be a causative agent in the development of cravings and feelings of loss of control. Children with ADHD exhibit abnormalities in the aforementioned systems, along with increased sensitivity to stress. This makes them most susceptible to smoking onset during their early years, as well as to the development of substance dependence later in life.

A lot of research has identified an association between early experimentation with psychoactive substances, and the subsequent development of substance use disorder (SUD) in adults diagnosed with ADHD. Longitudinal study [59] from childhood to adulthood have demonstrated that the use of illicit substances before the age of 15 significantly increases the likelihood of developing SUD in adulthood, independent of behavioral issues [59]. The pharmacological treatment of ADHD symptoms based on self-medication has been demonstrated to further increase the risk of alcohol and drug addiction [60]. The symptoms of ADHD in adults with SUD may be developmentally related to the symptoms and behaviors exhibited by children, particularly in cases of inattention, hyperactivity, and impulsivity [61], which are considered the primary symptoms of ADHD [62]. In addition to the elevated risk of alcohol, nicotine, or substance use disorders in ADHD, there are studies demonstrating the presence of an increased risk of other addictions of a behavioral nature, including gambling [63,64], gaming [65,66], and internet addiction [67]. The results of studies on Internet use indicated high scores on the so-called “CIUS” (Compulsive Internet Use Scale) in individuals declaring a confirmed diagnosis of ADHD, indicating a higher possibility of addiction in such cases [65,66]. Moreover, studies demonstrate that typical ADHD symptoms—impulsivity, restlessness, and compulsivity—are significant factors that increase the risk of internet addiction and even confirm a positive correlation between the presence of impulsivity and Internet addiction in individuals with ADHD [68,69]. In summary, behaviors such as impulsivity or compulsivity in ADHD are important symptoms that increase the risk of numerous, sometimes non-obvious addictions.

## 4. ADHD Psychopharmacotherapy

### 4.1. A Modern Approach to ADHD

Comprehensive initial evaluation of the core symptoms of ADHD, the presence of comorbidities, the patient’s age, their past treatment history, and the patient’s or family’s preferences should be considered before commencing therapy. It is important during treatment to reassess the presence of symptoms and side effects development over cycles of around 4–6 weeks. This is made possible by timely dosage adjustment, therapeutic modality change, or adding non-pharmacological interventions when needed. It relies on the presumption that treatment must be maintained as both effective and individualized according to the evolving needs of each patient. The efficacy of ADHD treatment is contingent upon the individualization of the therapeutic approach, the identification of risk factors, and the selection of therapeutic interventions [70,71,72,73]. A comprehensive approach to patients facilitates the implementation of an appropriate therapeutic regimen, with the potential to yield optimal long-term outcomes [74].

The onset of ADHD tends to emerge during early developmental stages. The age of onset has been documented to vary from one country to another. On a European scale, the earliest manifestations of ADHD delineated by the DSM guidelines, namely inattention, hyperactivity, impulsivity, were observed in the Netherlands at an average age of just over 2 years. By contrast, the latest manifestations were observed in Finland at an average age of 7.5 years [5].

Following a diagnosis of ADHD and the determination of an optimal treatment plan, it is important to acknowledge that treatment will frequently necessitate a lifelong commitment. For adults diagnosed with ADHD at an early stage of development, treatment frequently entails the continuation of therapy initiated during childhood [74].

**Figure 1:**
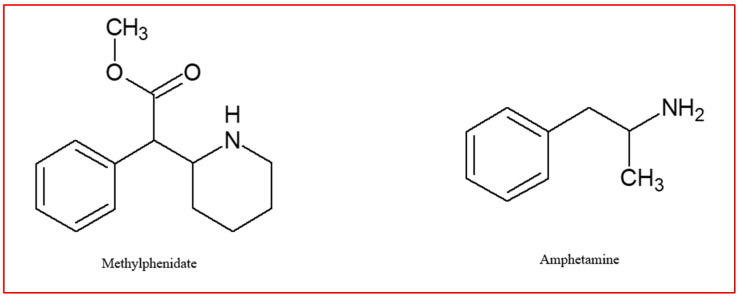
Stimulants used in the ADHD therapy.

**Figure 2:**
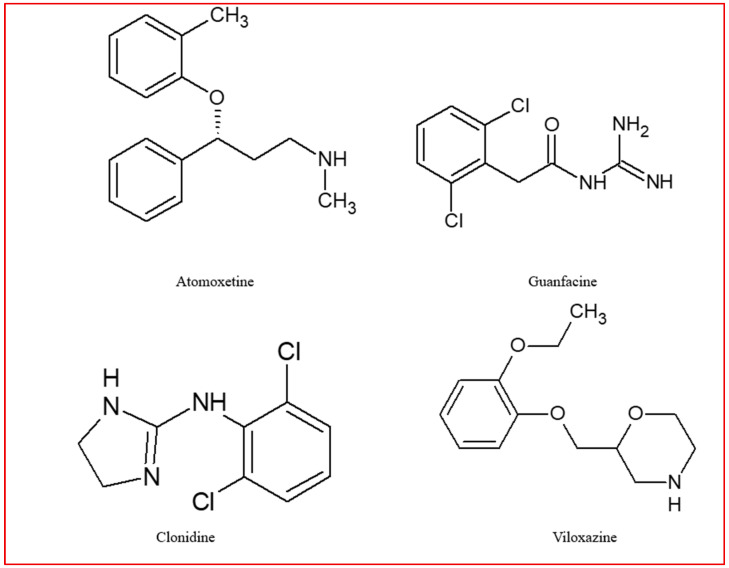
Non-stimulants used in the ADHD therapy.

At present, the management of ADHD treatment is comparable across different regions globally. In the majority of cases, psychoeducation is recommended as a preliminary measure. This involves informing the patient about the newly diagnosed condition and providing strategies for coping with it. Subsequent to this, long-term treatment is initiated, with the treatment plan being tailored to the patient’s current needs [74,75]. The therapeutic agents employed in the pharmacotherapy of ADHD are classified into two primary categories: psychostimulants and non-stimulants. The psychostimulants encompass methylphenidate (MPH) and amphetamines, while the non-stimulants include atomoxetine, guanfacine, clonidine, and viloxazine [25]. The utilization of clonidine and guanfacine is not advised in adults due to a paucity of research regarding their effectiveness and adverse effects in this age group [76]. Their structural formulas are presented in Figure 1 (stimulants) and Figure 2 (non-stimulants).

Charles Bradley first documented the efficacy of psychostimulants in the treatment of ADHD in 1937. His findings during the administration of Benzedrine (amphetamine) to children showed their improvement in school performance [77]. Sixty-two years later, findings from the MTA study confirmed the hypothesis that stimulants are more effective than behavioral treatment alone [78]. Atomoxetine has also demonstrated significant efficacy in randomized controlled trials [79]. Guanfacine, a post-synaptic α2A-adrenergic receptor agonist that was originally developed as an antihypertensive, was later used to reduce hyperactivity and impulsivity in children and adolescents with ADHD [80].

The mechanisms of action of the various ADHD drugs differ in several ways. These include the manner and magnitude of their effects on the body, their capacity to antagonize and agonize receptors and transport proteins in the central nervous system, and their ability to remodel neuronal networks in the brain [81].

The primary objective of the majority of medications employed in the pharmacotherapy of ADHD, particularly psychostimulants, is to augment the levels of catecholamines (dopamine and norepinephrine) within synaptic clefts [81]. The process is facilitated by the blockade of reuptake channels, specifically the DAT and NET channels for dopamine and norepinephrine, respectively [82]. Clonidine and guanfacine are classified as α2 receptor agonists, which enhances noradrenergic signaling within the brain. Metabolized bupropion is a norepinephrine reuptake inhibitor [81].

It has been found that psychostimulants have been linked to enhancement of attention and executive control, and most of the evidence supports such an association throughout life. These drugs elevate catecholamine tone in the fronto-striatal network for the purpose of maximizing the signal-to-noise ratio of the prefrontal cortex [76,83,84]. Psychostimulants also have the effect of enhancing concentration and self-control in individuals with ADHD, and it is observed across all ages. The mechanism of how these drugs exert their effect is through raising the levels of dopamine and norepinephrine in regions of the brain involved in decision-making and attention. This, consequently, results in better signal transmission within the prefrontal cortex, hence facilitating more efficient processing of information. The effect of aerobic exercises on inhibition/attention response has been depicted as low to moderate. This is possible by short-term catecholaminergic activation [85,86,87]. Exercise is a good adjunct, providing short-term enhancement in inhibition and response to attention after isolated sessions. Of additional interest is the fact that medication itself can create greater and more transferable gains in a variety of many different environments (e.g., home, school) as well as exercises [85,88].

Despite the variations in the mechanisms of action among individual ADHD medications, their effects are analogous across different medication groups. Increased concentrations of catecholamines in the synaptic space of brain neurons cause changes in the action of other neurotransmitters, including GABA and glutamate. These changes, in turn, modify brain areas responsible for cognitive function, the reward system, and memory [81].

Presently, the utilization of psychostimulants predominates in the domain of pharmacotherapy. Nonetheless, the employment of such compounds raises the likelihood of developing dependency and encountering an overdose [89].

It is important to point out that psychostimulants have several adverse side-effects to the body. Short-term physical effects of psychostimulants that users often perceive as positive are increased heart rate, blood pressure, and respiration. Psychologically, users become euphoric, more alert, and more self-assured. Withdrawal symptoms are primarily fatigue, dysphoria, increased appetite, insomnia or hypersomnia and psychomotor impairment [90]. Relatively harmless long-term side effects of treating ADHD with methylphenidate are predominantly loss of appetite, xerostomia and heart palpitations [91]. There are also more harmful side-effects of methylphenidate such as myocardial infarction, reversible ischemic stroke and psychotic episodes [92]. Psychostimulants also increase the risk of sudden cardiac death [93].

### 4.2. Methylphenidate

Methylphenidate (MPH) is a stimulant that acts on the central nervous system and is often the first-line psychopharmacologic treatment for ADHD [84]. In 1955, the Food and Drug Administration approved MPH for medical use within the United States [94]. Since that time, the quantity of the pharmaceutical agent prescribed has continued to increase. From 2010 and 2020 alone, the number of MPH users in the US quadrupled [95]. A similar growth trend has been observed in other regions worldwide, including those in Norway, the United Kingdom and the Netherlands [96,97,98].

**Figure 3:**
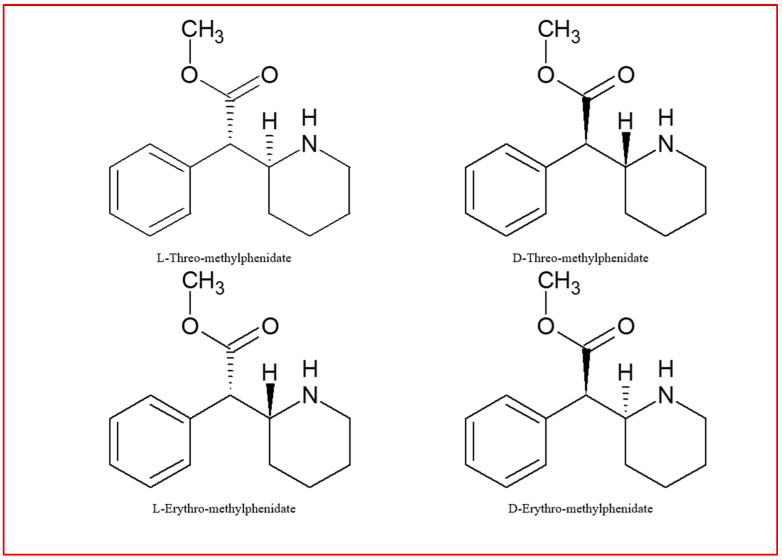
Different stereoisomers of methylphenidate.

MPH exists as four stereoisomers (dextro/levo-treo/erythro) that differ in their biological activity (as shown in Figure 3). It has been demonstrated that only the treo forms affect the central nervous system, and among these forms, dextro-treo-methylphenidate (d-MPH) is the sole compound capable of traversing the blood–brain barrier [82].

Methylphenidate is a lipophilic substance, with a mere 15% of the compound being bound to proteins [99]. This results in rapid distribution of the drug in the tissues, as well as a rapid effect of the drug. The maximum concentration of MPH is generally attained between one and three hours following the ingestion of an instant-release formulation. The observed discrepancies can be attributed to a variety of individual factors, including age, gender, and dietary habits [82].

Presently, MPH is available in several forms. These agents differ in several ways, including the manner of administration, the mechanism of release, and the duration of their activity. It is imperative to select the appropriate pharmaceutical agent for the patient’s current requirements, while considering the contraindications associated with each formulation of the drug [100]. It is imperative to acknowledge the potential consequences of MPH administration, including the induction of hyperglycemia and the subsequent reduction in blood potassium levels. This, in turn, can result in the occurrence of torsade de pointes ventricular tachycardia [101].

The earliest iteration of the MPH pharmaceutical compound is characterized as an instant-release formulation. Due to its brief duration of action, the medication must be ingested multiple times per day. This necessity arises from its minimal half-life (T_1/2_ = 2.9 h) [102]. This results in substantial daily fluctuations in plasma concentrations, which can lead to adverse health effects, including irritability, mood swings, and cognitive impairment [82,100]. Furthermore, it has been demonstrated that this phenomenon concomitantly increases the risk of abuse [103]. The development of modified MPH release methods has emerged as a response to the need to counteract this phenomenon [82,100].

One of the earliest examples of this approach was the osmotic-controlled release oral delivery system (OROS) [100]. In its simplest form, this type of drug consists of two compartments surrounded by a semi-permeable membrane. One of these containers serves as a storage medium for the drug, while the other is employed to hold a water-swollen compound. Subsequent to ingestion, the OROS-type drug traverses the digestive system, where it undergoes osmosis and absorbs water. This process culminates in the drug’s expulsion from the compartment through a meticulously designed opening [104]. The advent of this technology has led to a marked increase in efficacy and a concomitant reduction in the amount of MPH consumed per day, with the consumption now limited to a single serving of the drug [100].

Subsequent technological advancements have further refined the drug’s delivery formula, enhancing its effectiveness and prolonging its duration of action, while concomitantly reducing the risk of abuse and addiction [100]. Furthermore, pharmaceutical agents were engineered in a form that could not be crushed for the purpose of dissolving the active ingredient and delivering it intravenously or intranasally [105].

One such method is the delivery of MPH by absorption through the skin via specialized transdermal patches. The employment of this method facilitates the modulation of the duration of the pharmaceutical agent’s effect on an individual by merely removing the adhesive patch, thereby enabling pharmacotherapy for patients afflicted with gastrointestinal maladies and dysphagia [12]. The drug is recommended to be applied on the hip for no more than 9 h. During the wear time MPH reaches its maximum blood concentration. After removal of the patch the half-life of methylphenidate equals between 3 and 4 h [106].

The drug is absorbed directly into the bloodstream, thus circumventing the absorption of the active substance through the intestinal epithelium and averting potential irritation. It has also been demonstrated to impede the elevated first-pass metabolism in liver cells, thereby exhibiting hepatoprotective properties [107].

In their study, McRae-Clark et al. [108] demonstrated that the administration of methylphenidate (MPH) via the skin (transdermal) led to a reduction in stimulant abuse. The study’s participants included 14 adults diagnosed with ADHD, including six males and eight females, aged between 18 and 65, with a history of abuse and addiction to stimulants. Prior to the study, half of the subjects had previously abused medications prescribed by a physician. The patients were administered eight weeks of transdermal MPH therapy. Patients were required to report for follow-up and undergo drug testing on a weekly basis to detect the presence of narcotics in their urine. The study’s findings revealed that none of the 107 urine samples collected tested positive for stimulant substances. However, the presence of other substances, including marijuana, opioids, and benzodiazepines, was detected in five samples. The results of the study suggest that the administration of transdermal methylphenidate therapy may contribute to the prevention of potential abuse of stimulants during pharmacotherapy for ADHD. However, it is imperative to acknowledge that this was an open study with a limited number of participants. Consequently, the findings may not be representative of studies with a larger population [108].

Mentioned in this text forms of MPH implemented in ADHD therapies and their beneficial role are collected in Table 2.

### 4.3. Amphetamines

Amphetamines represent another class of stimulant medication utilized in the treatment of ADHD. These include dextroamphetamine, which is the right-handed enantiomer of amphetamine, amphetamine racemate salts and lisdexamphetamine [109].

The aforementioned compounds have the capacity to influence dopamine and norepinephrine transporters. Moreover, they possess the ability to induce the release of monoamines from synaptic cilia and to inhibit monoamine oxidases (MAO). This results in an increase in the amount of neurotransmitter in the synaptic space [82].

Chemically, amphetamines are classified as sympathomimetic amines [110]. These substances have been demonstrated to exert significant hepatotoxic effects. Consequently, it is imperative to take the hepatic condition of the patient into account when prescribing this particular pharmacotherapy. Complications of an undesired nature may encompass acute liver failure and hepatitis [111].

Amphetamines have also been demonstrated to possess psychoactive properties. These substances have been demonstrated to induce feelings of euphoria and pleasure, while also exhibiting strong addictive effects. This has resulted in their classification as psychotropic substances intended for non-medical applications [112].

Two enantiomers of amphetamine have been identified in nature: the dextro form (dextrorotatory, d-AMP) and the levo form (levorotatory, l-AMP). The two substances have been demonstrated to possess biological activity, including the capacity to enhance the excretion of monoamines. The primary distinction between these two categories lies in the specific monoamine secretion they stimulate. It has been demonstrated that D-AMP exerts a greater effect on dopamine, while the opposite is true of l-AMP with respect to norepinephrine. It has also been demonstrated that the half-life of d-AMP is shorter than that of the other compound [113].

For medical purposes, amphetamine is available in various formulations. The medication is available in both immediate-release and extended- or modified-release formulations, with the option of transdermal patches as well. A comparison of these forms with those used for methylphenidate reveals no significant disparities in either the release mechanism of the active ingredient or the benefits of their use [100].

A salient property employed in the treatment of amphetamine, as exemplified by its use in mixed amphetamine salts, pertains to the observation that the biological half-life of both enantiomers is augmented when they are present in the body concurrently [113]. This phenomenon can be attributed to the observation that both d-AMP and l-AMP are subject to metabolism by cytochrome CYP2D6. However, d-AMP exhibits a higher binding affinity, resulting in a prolonged residence time for l-AMP within the circulation. Consequently, this prolonged exposure to l-AMP leads to a prolonged duration of effect on the nervous system [83]. Therefore, both racemic and non-racemic mixtures are utilized, exhibiting varying durations of action due to their distinct compositions [113].

Another amphetamine employed in the treatment of ADHD is lisdexamphetamine (LDX). It is a prodrug that is metabolized in the body to d-AMP and lysine [114,115]. This approach is gaining traction within the domain of pharmacotherapies. In Australia, as of 2022, the drug has been the top-selling medication for ADHD [116].

LDX exhibits an interesting property: namely, that it does not undergo biotransformation in the intestines. Following absorption into the bloodstream, the substance undergoes transformation under the influence of erythrocytes [114]. LDX lacks the capacity to traverse the blood–brain barrier; it only becomes biologically active after undergoing metabolic conversion to its active form. This results in a delay in the onset of action and a sustained release of the active ingredient [82]. Concurrently, this should lead to a reduction in the addictive potential of lisdexamphetamine [117]. This theory was proposed by David W. Goodman in 2007, and it was derived from an analysis of two clinical trials [118].

The first study was conducted on a sample of 38 adults with a documented history of stimulant abuse, of which only 36 participants completed the study. Patients were administered either 40 mg of d-AMP or LDX in doses of 50, 100, or 150 mg. The addictive potential of the administered substance was subsequently assessed by means of a series of inquiries designed to elicit a subjective rating of the subject’s current state of mind on a scale ranging from 1 to 29. It was observed that as the LDX dose increased, so did the addictive potential. The potential of a 100 mg dose was found to be significantly lower than that of a 40 mg dose of d-AMP. 150 mg of LDX demonstrated a level of efficacy that nearly matched the administered amphetamine dose [119].

The second study was conducted on a sample of nine adults with a history of addiction. Patients were administered one of three substances: d-amphetamine sulfate, lisdexamphetamine, or placebo. The maximum blood concentrations of the active substance were obtained more than 1.5 h later with LDX than with d-AMP. The addictive potential of the administered substances was also determined through the subjective responses of the subjects to the questions asked. The addictive potential score obtained for LDX was not statistically different from the placebo [120].

These findings led David W. Goodman to formulate the hypothesis that LDX exhibits reduced addictive potential due to its role as a prodrug, which undergoes a biotransformation process to become an active substance. Furthermore, LDX attains lower blood concentrations in comparison to d-AMP of equivalent mass [118]. It is noteworthy that both studies, conducted by the same research team, did not include individuals diagnosed with ADHD. The findings of this study may not be indicative of the entire patient population [119,120].

The hypothesis proposed by Goodman [118] has not been corroborated by the findings of Wolfgang Kaemmerer’s 2024 paper. The findings indicate that the likelihood of developing an addiction to this medication is comparable to that of immediate-release dexamphetamine, suggesting that the onset of addiction is merely postponed [115]. The conclusions arrived at were derived from the same research work as Goodman’s [115,118]. Moreover, the 2017 study by Dolder et al. [121] was considered, wherein participants were administered 40 mg of d-AMP or 100 mg of LDX, followed by the assessment of blood concentrations of the active ingredient. The concentrations of the active substance for LDX were equivalent to those for d-AMP; however, they occurred one hour later. The same relationship was obtained in measuring addictive potential by asking participants how they felt on a scale from 0 to 100 [121]. In the light of these studies, Kaemmerer proposes that the addictive potential of lisdexamphetamine remains unabated in comparison to its equimolar counterpart of d-AMP, with the distinction being the temporal location of the drug’s effect [115].

It is noteworthy that a prodrug serdexmethylphenidate (SDX) is available in the pharmaceutical market. It undergoes biotransformation to methylphenidate. In 2021, the FDA approved the medication for the treatment of ADHD in children aged six years and older [122]. It has been demonstrated that SDX exhibits a high degree of permeability into the bloodstream in the initial sections of the small intestine. However, the prodrug exhibits low binding affinity for erythrocytes, leading to its inefficient metabolism into the active product within blood vessels. Consequently, it is excreted into the large intestine for biotransformation, and subsequently reabsorbed. The result of these factors is that SDX has minimal utility in the treatment of ADHD. To enhance its efficacy, it must be coated with instant-release methylphenidate [114]

**Figure 4:**
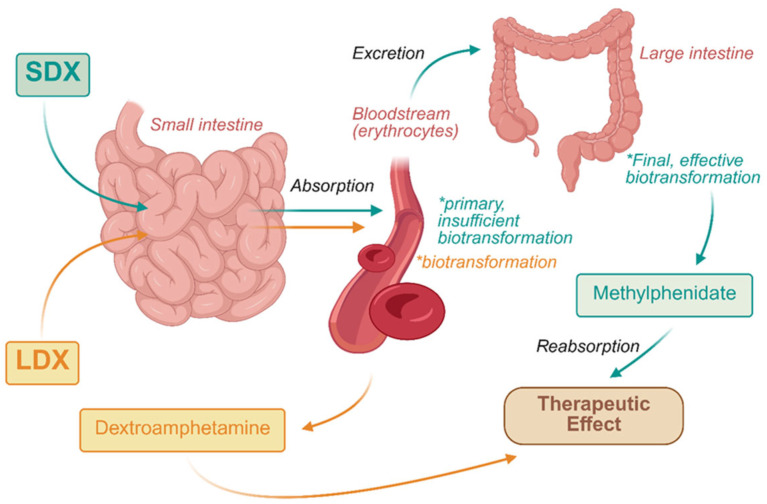
LDX and SDX—sites of metabolism and transport in the human body. In the figure, “*” denotes a process. Graph was prepared using Biorender. Żełabowski, K. (2025) https://BioRender.com/4ad64bp (accessed on 29 July 2025).

Biotransformation of LDX and SDX are presented in Figure 4.

### 4.4. Dopaminergic Psychostimulants and the Risk of Addiction

The prevalence of addiction in individuals diagnosed with ADHD constitutes a grave global concern. Rohner et al. [123] have indicated that as many as 21% of individuals grappling with addiction are also diagnosed with ADHD. Furthermore, the onset of abuse in this population is typically more rapid compared to the general population. It is imperative to identify the most effective strategy for addressing this issue [123].

The use of stimulants, which are addictive compounds, raises significant concerns. This has led to concerns regarding the potential for misuse of these drugs for non-medical purposes, as well as the use of these compounds in doses and intervals that are not recommended [89]. In Australia, between the years 2000 and 2024, a total of 37 individuals diagnosed with ADHD lost their lives to overdoses of stimulant medications, including MPH, LDX, and d-AMP. Notably, 15 of these fatal overdoses occurred in instances where the stimulant medications in question had been prescribed by a medical professional [124].

One of the methods of dealing with the risk of addiction, in addition to selecting appropriate pharmacotherapy, is the introduction of so-called “drug vacations.” In such cases, patients are closely monitored by medical professionals while undergoing a period of drug abstinence. Subsequently, the patient’s vital signs are meticulously monitored to ascertain the necessity of continued treatment. This also serves to impede the development of tolerance to the active substance [125]. Prolonged utilization of pharmaceutical agents that facilitate dopamine secretion within the nervous system has been demonstrated to induce a downregulation, characterized by a decline in the number of receptors present on the postsynaptic membrane. The practice known as “drug holidays” has been demonstrated to facilitate the reconstruction of the number of receptors [126].

In order to qualify for the “drug holidays”, patients are required to meet the following criteria. Firstly, the treatment they have been undergoing must be persistent for the prolonged period of time. Secondly, if patients had the “drug holidays” before, it could not have caused any negative consequences. Thirdly, during the “drug vacations” patients should have time to concentrate and check for any remission [127]. It is recommended that patient should undergo the “drug holidays” during periods of time when it wouldn’t deteriorate his functioning, for example, during summer vacations or long weekends [128].

From April 2018 to February 2023, a cohort study was conducted by Molina et al. [129]. The objective of the study was to analyze the impact of stimulant use on subsequent substance abuse. Between 1994 and 1996, subjects were selected from a total of six sites in the United States and one in Canada, with the subjects being children between the ages of 7 and 9. Subsequently, the subjects were observed at regular intervals to ascertain whether they had engaged in the adverse use of various substances, including alcohol, cannabinoids, and nicotine. The researchers examined a total of 579 children using this methodology. The study revealed that initially, the number of individuals consuming such substances increases with age. However, in the early adult years, the percentage of such users stabilizes and begins to remain constant, no longer changing with age. However, this trend is also observable among individuals without ADHD who do not utilize psychostimulants for treatment purposes. The analysis of this cohort study concluded that pharmacotherapy with stimulants does not have a negative or positive effect on the percentage of people who choose stimulants. A limitation of the study is the absence of medical history and thus the researchers relied exclusively on the parents’ reports [129].

A randomized controlled clinical trial was published in 2018 by Frances R. Levin et al. The objective of the study was to determine the impact of pharmacotherapy with amphetamine salts on cocaine addiction in patients diagnosed with ADHD [130]. The present analysis was based on a previously published paper by the same research team, which described the data collected in meticulous detail [131]. The study examined 126 participants aged 18–60 over a 14-week period, giving them either a placebo or 60 mg or 80 mg daily doses of AMP salt. The experiment was conducted in a double-blind manner, meaning that neither the investigators nor the subjects were aware of whether a placebo or the pharmaceutical agent had been administered. Patients were required to appear at the clinic three times per week to collect urine samples for cocaine tests. To obtain statistical data, the researchers treated each week in which cocaine was detected in a minimum of one of two samples collected as cocaine-positive weeks. Furthermore, weeks in which participants self-reported cocaine use and weeks with insufficiently collected data were also designated as cocaine-positive weeks [130,131]. The study’s findings indicated that the probability of achieving abstinence was approximately five times higher in subjects who received 80 mg of AMP compared to those who received a placebo. The odds ratio for the 60 mg amphetamine salt dose was determined to be 2.92 [131].

It is noteworthy that positive results were also obtained in subjects without ADHD. A meta-analysis of 38 clinical trials by Vitor S. Tardelli et al. demonstrated that the administration of AMP results in prolonged abstinence time and an augmented number of days during which subjects do not use cocaine [132].

The underlying mechanism that precipitates this phenomenon is not yet fully understood. One hypothesis posits that amphetamine and cocaine exert their effects on dopamine transporters of a similar type. AMP would then block the effects of cocaine, consequently inhibiting ability to achieve desirable effect. The long-release version would prolong this condition [133]. A further observation is that individuals who ingest prescribed stimulants do not subsequently express a desire to seek them out on their own accord [118].

The available literature does not indicate any negative or positive effects of AMP use on marijuana addiction [134]. A significant decrease in cases of nicotine dependence was observed in patients with ADHD who were treated with long-term pharmacotherapy, primarily MPH, but also d-AMP [135].

A study by Ezard et al. [136] on the effect of LDX on methamphetamine addiction showed positive results during the first 12 weeks of therapy. A marked decline was observed in the number of days on which patients used methamphetamine [136].

In contrast, Acheson et al. [137] propose a contradictory hypothesis, suggesting that the administration of lisdexamphetamine to methamphetamine-dependent patients would enable them to better withstand the withdrawal syndrome phase, including through the stabilization of vital signs and the reduction in psychotic episodes. To date, this theory remains unproven and requires further validation [137].

In summary, the majority of studies on the impact of stimulant pharmacotherapy on addiction have demonstrated that the administration of amphetamine, dextroamphetamine, or methamphetamine, among other substances, does not result in adverse effects on substance use disorder. In fact, these medications can facilitate the process of abstinence by enabling patients to discontinue the use of addictive substances [105,135,138,139]. It is evident that patients who use pharmacotherapy that includes stimulants demonstrate enhanced resilience during the withdrawal phase from addictive substances [137].

### 4.5. Non-Stimulants in ADHD Therapy

#### 4.5.1. Atomoxetine

The utilization of stimulants as the primary pharmacological intervention for ADHD represents a prevailing therapeutic approach. Nevertheless, there are instances in which such pharmaceutical interventions do not yield the anticipated outcomes. Not all patients tolerate the therapies properly, or adverse effects occur, including cardiovascular problems, which undermine the potential of long-term pharmacotherapy. For some individuals, the potential for developing an addiction or an existing substance use disorder renders the continuation of stimulant pharmacotherapy impracticable. In such cases, the patient should be referred to treatment with non-stimulants or to combination therapy consisting of a lower dose of stimulants and non-stimulants [140].

At present, only four non-stimulants have been approved for the treatment of ADHD: atomoxetine (ATX), clonidine, guanfacine, and, since 2021, viloxazine [141]. ATX and viloxazine function as norepinephrine reuptake inhibitors (NRI), while clonidine and guanfacine act as α2 adrenergic receptor agonists (α2ARAs) [142]. A preliminary analysis reveals that only one of these four medications, ATX, has undergone testing to assess its efficacy in addressing addiction in patients diagnosed with ADHD [143].

ATX exhibits a high degree of affinity for norepinephrine transporters located on the presynaptic membrane. Consequently, ATX selectively binds to these transporters, impeding the reuptake of norepinephrine. This results in an increase in the concentration of this neurotransmitter within the synaptic space. The affinity for transporters of other neurotransmitters, such as dopamine or serotonin, is negligible [144].

In 2010, Thurstone et al. [145] conducted a randomized clinical trial to assess the effects of atomoxetine on ADHD and dependence syndrome. The study’s participants were between the ages of 13 and 19, totaling 70 individuals. Half of the participants were administered ATX, while the remainder received a placebo. The drug and placebo were administered for a period of 12 weeks, during which patients were asked to report the number of days in the previous 28 on which they had taken the substance to which they had developed an addiction. The study did not address the issue of nicotine addiction. The study’s findings indicated that the duration of abstinence from addictive substances was comparable between the ATX and placebo groups. The findings indicate that the prescription of atomoxetine to patients diagnosed with ADHD who also meet criteria for substance use disorder will not result in the exacerbation of their underlying condition [145].

Atomoxetine pharmacotherapy necessitates constant monitoring. In the United States of America and Australia, among other countries, ATX has been identified as a substance that may potentially increase the risk of suicide [146]. The systematic review conducted on this topic does not support this theory. Kim et al. [147] included nine studies to date, thus covering 81,739 patients taking ATX. The researchers observed no significant differences between the ATX and control groups, indicating that atomoxetine use did not appear to influence the increase in suicide attempts [147].

Atomoxetine has demonstrated positive effects in patients with comorbid anxiety conditions. The treatment has been shown to have a positive effect on the patients, with symptoms being reduced [148,149].

#### 4.5.2. Bupropion

**Figure 5:**
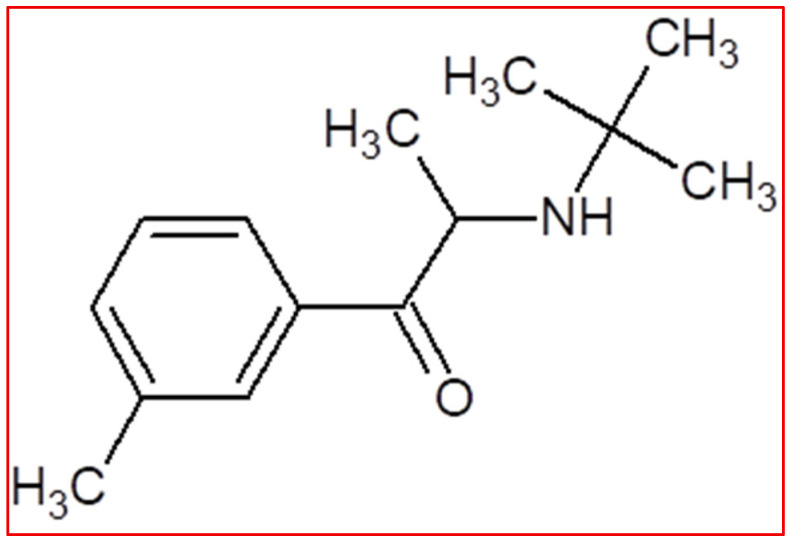
Chemical structure of bupropion.

Bupropion (its structural formula is presented on Figure 5), an antidepressant pharmaceutical agent, has also received approval for the treatment of seasonal affective disorder and nicotine dependence [150]. In the United States, as of 2021, it is ranked the third among antidepressant medications prescribed by healthcare professionals, following sertraline and trazodone. Moreover, it was previously the antidepressant drug with the highest number of expenditures by US Medicaid until 2019, when the record was surpassed by vortioxetine [151].

Since the approval of bupropion for the treatment of depression in 1985, it has also been prescribed for other conditions, employing off-label utilization. Such conditions include sexual dysfunction caused by antidepressant use, obesity or ADHD [150].

The mechanism of action of bupropion, which is distinct from that of other antidepressants, suggests its potential as a treatment for ADHD. Bupropion lacks serotonergic properties. Instead, it exerts its effects on monoamine transporters (dopamine and norepinephrine), thereby impeding the reabsorption of these neurotransmitters. Consequently, it is evident that the medication does not elicit adverse effects that are associated with other antidepressants, including weight gain, lethargy, or sexual dysfunction [152].

The efficacy of bupropion for the treatment of ADHD remains to be definitively established. In a review, Verbeeck et al. [153] included six papers available at that point (five American and one Iranian) conducted on adults. The objective of the analysis was to ascertain the efficacy of bupropion in the treatment of ADHD. However, the available data are insufficient to reach a definitive conclusion on the matter. The studies demonstrated patient improvement in comparison to the placebo group; however, they were characterized by the risk of bias on the part of the study conductors. Two of the studies were funded by pharmaceutical companies, and in one study, the lead author was employed as a consultant for pharmaceutical companies. It was also impossible to demonstrate the long-term effects of such therapy. Furthermore, the studies excluded individuals with coexisting substance use disorder [153].

Riggs et al. [154] conducted an open-label study that examined the effects of five weeks of bupropion pharmacotherapy in 13 boys with ADHD who also met criteria for a co-occurring substance use disorder. Following the designated time period, the collected scores were subjected to analysis, employing the Conners scale method for the purpose of evaluation. Additionally, urine samples were obtained to ascertain the presence of addictive substances. The presence of narcotics was detected in the urine samples of two patients: amphetamines in one patient and tetrahydrocannabinol in the other. The study revealed that patients who received bupropion exhibited significant improvements. The limitations of this study are as follows: the number of participants is small; there is a lack of follow-up trials; results are only collected after the completion of pharmacotherapy; and there is a lack of better monitoring of addiction status [154].

The study by Solhkhah et al. [155] corroborated the findings previously obtained by Riggs et al. [154], while concurrently augmenting our extant knowledge on the subject. The present study involved 14 subjects of both sexes with diagnosed ADHD and affective disorder who were addicted to at least one non-nicotine substance. The subjects took a daily dose of bupropion for 3 to 6 months, starting with 100 mg, which was gradually increased over the course of the experiment. The maximum dose administered was 400 mg. The collection of results involved the utilization of the DUSI scale. The experimental results were positive. There was an improvement in the patients’ condition. In a majority of the subjects, a reduction in the use of addictive substances was observed. This reduction was statistically significant in 93% of the subjects when compared to the placebo [155].

When prescribing bupropion, it is imperative to exercise caution, particularly in cases of known epilepsy. Bupropion has been demonstrated to elicit seizures of an epileptic nature [156]. Moreover, it is imperative to avoid concomitant administration of clozapine, as it can potentially induce seizures in patients who have not exhibited such reactions previously [157].

## 5. Non-Pharmacological Treatment

The treatment of ADHD in adults should be multifaceted. The optimal outcomes are attained through the integration of pharmacological and non-pharmacological approaches [74]. In addition to psychoeducation, non-pharmacological methods encompass behavioral therapy and modifications to the patient’s environment and lifestyle [74,75].

The following alterations are recommended: establishment of a daily routine that includes organization of a sleep schedule and the completion of professional obligations. This can also take the form of determining where important items, such as keys or a wallet, should be placed for easier retrieval. Furthermore, a potentially advantageous approach involves the implementation of a system utilizing colored cards to facilitate expeditious retrieval of significant documentation, notably medical records [158].

Non-pharmacological methods are particularly recommended for children, and pharmacotherapy should only be prescribed in special cases [159]. For individuals under the age of 17, stimulants, as a primary pharmacological treatment methylphenidate and amphetamines are recommended. In instances where initial therapeutic interventions have not yielded the desired outcomes, non-stimulant medications are considered a secondary treatment option [9].

### 5.1. Cognitive-Behavioral Therapy (CBT)

CBT is well-known non-pharmacological treatment for ADHD, particularly effective when combined with parental involvement or medication. It is based on instrumental conditioning, a behavioural approach that uses reinforcement and punishment to shape desired behaviours. This includes positive reinforcement (adding a rewarding stimulus after correct behaviour) and negative reinforcement (removing an unpleasant stimulus). Likewise, positive punishment (adding a negative stimulus after incorrect behaviour) and negative punishment (removing a positive stimulus) are also applied to discourage unwanted actions [160].

### 5.2. Dietary Interventions and Supplementation

Studies on dietary supplements in ADHD have shown mixed results [161,162,163]. Randomized controlled trials have examined substances like amino acids, minerals, vitamin D, melatonin, and phosphatidylserine [164,165,166], with some evidence supporting the use of fatty acids in children [165]. However, findings on polyunsaturated fatty acids are inconsistent and show no significant improvement [167]. While supplements should not replace standard treatments, they may serve as a safe and supportive addition, potentially reducing medication side effects [167].

### 5.3. Neurofeedback

Neurofeedback is a non-pharmacological method that trains individuals to self-regulate brain activity using real-time EEG feedback through a brain–computer interface [168]. It aims to increase beta activity and reduce theta waves, thereby improving attention and behaviour [168]. A meta-analysis of 14 studies (2004–2021) involving 718 participants showed short-term efficacy in reducing ADHD symptoms [168]. However, a rigorous randomized controlled trial found no significant difference between real and placebo neurofeedback [168] and comparisons with other treatments (e.g., CBT, cognitive training, medication) also showed no clear superiority [168,169].

### 5.4. Brain Stimulation

Brain stimulation techniques, such as trigeminal nerve stimulation (TNS), transcranial magnetic stimulation (rTMS) [170,171,172], and direct current stimulation (tDCS), have been explored as novel ADHD treatments. The Monarch^®^ TNS device was approved by the FDA in 2019 for children aged 7–12 not taking medication [173]. However, due to limited blinded trials and insufficient evidence, brain stimulation is not currently recommended as a standard ADHD treatment [160,174,175].

### 5.5. Music Therapy

Music therapy—in active (playing), passive (listening), or interactive (rhythmic exercises) forms—has shown potential to improve attention, self-regulation, academic performance, and reduce impulsivity and aggression. Effects depend on the form and type of music, with calming music promoting relaxation and better autonomic regulation, while rhythmic exercises support cognitive and emotional functions [176].

### 5.6. Physical Exercise

Studies have established that exercise provides modest yet significant benefits for the patients with ADHD. Single aerobic session improves the attention and response inhibition ability, possibly through short-term catecholaminergic activation [85,86]. Moreover, there is a proof that exercise can help with reducing anxiety, depression, and emotional dysregulation in children with ADHD [177].

## 6. Discussion

Our comprehensive review of the extant literature reveals a need for a holistic approach to the treatment of patients with ADHD and co-occurring addictions. The optimal therapeutic approach includes pharmacological and non-pharmacological interventions [74]. Non-pharmacological methods consist of behavioral therapy and environmental and lifestyle modifications, and they are recommended, especially for preschool children. Pharmacotherapy should be prescribed only in special cases [9,74,75]. Conversely, in young people under the age of 17, stimulants are recommended as a first-line treatment, with methylphenidate and amphetamines being the most commonly prescribed medications. Subsequently, the drugs under consideration are non-stimulants [9].

Non-pharmacological therapies, including cognitive-behavioral therapy, can serve as a valuable adjunct to pharmacotherapy, particularly in ADHD accompanied by anxiety disorders. According to the research, cognitive behavioral therapy has demonstrated moderate efficacy in reducing symptoms of ADHD and enhancing emotional functioning [160,178]. Despite the initial optimism fueled by promising results, controlled studies have revealed that neurofeedback and brain stimulation don’t demonstrate unequivocal effectiveness, necessitating further investigation [179,180].

It should be mentioned that CBT is also proven to be effective in adults, particularly with comorbid mood or anxiety symptoms. It helps them with stress management and emotional dysregulation [181,182]. In contrast, musical therapies appear to be more effective among children with ADHD, where structure through activities may prove to be an effective pathway in controlling attention. Combined with additional approaches such as yoga, they reduce inattention, hyperactivity/impulsivity, and oppositional behavior [183,184].

With regard to pharmacotherapy, the predominant approach involves the utilization of psychostimulants, such as methylphenidate, which is recognized as a first-line medication for the management of ADHD [84]. Additionally, the use of amphetamines is employed. However, this approach is associated with an elevated risk of developing addiction [89]. The amphetamine itself exhibits significant hepatotoxic effects, potentially resulting in adverse consequences such as acute hepatitis [89]. Therefore, it is important to consider the hepatic status of the patient when prescribing this particular pharmacotherapy [111]. Amphetamines induce feelings of euphoria and pleasure. Of particular significance is strong tendency to produce addictive effects, which leads to for non-medical purposes [112]. Conversely, a separate study on the use of amphetamines in the treatment of ADHD suggests a favorable outlook on the potential for combined therapy in managing patients with co-occurring cocaine addiction. This consideration is based on the premise that amphetamines—despite their potential for abuse—may offer therapeutic benefits in treating ADHD with comorbid cocaine dependence [130]. However, another study found that the number of people consuming such substances initially increases with age, and in early adulthood, the percentage of such people stops fluctuating and begins to stay at the same level, no longer changing with age. However, this trend is also observable among individuals without ADHD who do not use psychostimulants for treatment purposes. The findings of this cohort study demonstrate that the utilization of medication with stimulants doesn’t influence the proportion of individuals who opt for stimulants [129].

The majority of studies on the effects of psychostimulants on addiction have indicated that the use of methylphenidate and amphetamines doesn’t increase the risk of developing a substance use disorder. In fact, these substances may even assist in the treatment of SUDs by prolonging the period of abstinence and reducing the use of unwanted drugs [105,135,138,139]. Amphetamine has been the focus of particular research due to its suggested beneficial effects in reducing cocaine dependence in patients diagnosed with ADHD. A randomized, controlled clinical trial was conducted to determine the efficacy of amphetamine salt administration in promoting abstinence from cocaine. The study revealed that compared to placebo, amphetamine salt administration significantly increased the percentage of patients maintaining abstinence from cocaine [130]. The experiment was conducted under double-blind conditions, a methodological approach that was adopted to mitigate the potential for errors that may have been influenced by the expectations of the researchers and participants. Despite the fact that the manner in which this study was conducted led to a significant reduction in the number of potential errors, further studies are necessary to determine the long-term effects of this therapeutic strategy. Consequently, it is important to conduct additional studies to definitively ascertain the safety and efficacy of amphetamine as a therapeutic agent for patients with ADHD who also have comorbid addictions.

Atomoxetine, a non-psychostimulant drug, has been the subject of concern due to its potential association with an elevated risk of suicide [145]. This study suggests that the prescription of atomoxetine to patients with ADHD and a co-occurring addiction syndrome may not exacerbate their condition or increase the likelihood of suicide attempts. Furthermore, atomoxetine has positive effects on individuals diagnosed with co-occurring anxiety conditions. The medication to enhances the condition of patients, thereby reducing their symptoms [149]. These findings suggest that the medication may be a suitable treatment option for ADHD who are at risk of developing addiction.

A subject of future research that may prove to be of interest is bupropion, a substance that lacks the adverse effects in comparison to other antidepressants [152]. The efficacy of bupropion for the treatment of ADHD is still in the research phase, the number of which is insufficient to give a definitive opinion on its effectiveness. Although research demonstrated a significant improvement in patients’ conditions, with 93% of subjects experiencing a reduction in substance abuse and another study also showed an improvement in the condition of bupropion users [154]. The long-term effects of bupropion therapy have not been revealed. Despite the evident benefits observed in the patient’s condition in the studies conducted on this substance, when compared to the outcomes associated with the administration of a placebo, the impartiality of the researchers has not been confirmed. Furthermore, the long-term implications of such therapeutic interventions remain unexamined, and individuals afflicted with coexisting addictive disorders were systematically excluded from the studies [153].

All studies mentioned in this paper about use of ADHD and their impact on SUD were collected in Table 3.

## 7. Conclusions

ADHD is a chronic neurodevelopmental disorder that significantly impacts daily life, increasing the risk of educational, occupational, and social problems. Individuals diagnosed with ADHD have an increased likelihood of developing addictions, a phenomenon that can be attributed to their tendency towards impulsivity and deficits in behavioral regulation. In the treatment of substance use disorders, pharmacotherapy, particularly psychostimulants, has demonstrated the most efficacy. However, for patients with a history of addiction, medications with reduced addictive potential are recommended. Non-pharmacological therapies, including cognitive-behavioral therapy, are effective in reducing symptoms and enhancing social functioning. The treatment of ADHD as an individualized process involves the adaptation of therapeutic procedures based on the primary symptoms manifested, comorbidities that may exist, patient age, and patient or family preference. Reassessments must be conducted frequently every two months to be able to make adjustments in dosage, treatment type, or the inclusion of non-pharmacological approaches. This approach shows both safety and effectiveness without compromising the congruence of care with evolving patient needs. Further research is necessary to optimize treatment approaches, especially in the context of patients at increased risk.

## Figures and Tables

**Table 1:** Criteria of adult ADHD diagnosis accordant with ICD-11 and DSM-V.

Criteria	ICD-11	DSM-V
Persistent time of symptoms	6 months or longer	6 months or longer (in two different settings)
Inattention	Symptoms are persistent and have a direct negative impact on functioning, including: Difficulty in sustaining attention to tasks that do not provide a high level of stimulation Making careless mistakes in school or work assignments Not completing tasks Easily distracted by extraneous stimuli Often does not seem to listen when spoken to directly Loses things Forgetfulness in daily activities	Five or more symptoms including: Failure to give close attention to details Careless mistakes in schoolwork, at work, or with other activities Trouble with holding attention Seeming like non-listening when spoken to directly. No following through on instructions (loss of focus, side-track). Trouble with organizing tasks and activities. Often loses of things necessary for tasks and activities Forgetfulness in daily activities
Hyperactivity	Symptoms are persistent and direct negative impact on functioning, including: Excessive motor activity Leaving seat when expected to sit still Feelings of physical restlessness, a sense of discomfort with being quiet or sitting still Difficulty in engaging in activities quietly A tendency to act in response to immediate stimuli without deliberation or consideration of risks and consequences	Five or more symptoms, including: Often leaving seat in situations when remaining seated is expected Excessive speech patterns Often interruptions or intrusion on others Feelings of restlessness Often running about or climbs in situations where it is not appropriate Unable to play or take part in leisure activities quietly

**Table 2:** Methylphenidate application forms.

Form	The Manner of Application	Benefits of This Form of Therapy	Weakness of This Form of Therapy	Risk of Addiction
Instant-release	Absorption in digestive system (oral administration)	Instant way of action	Brief time of action, Must be ingested multiple times per day, Daily fluctuation of active compound (resulting in irritability, mood swings, and cognitive impairment), Increases the risk of abuse	High (under revision)
The osmotic-controlled release oral delivery system (OROS)	Oral administration Osmotic releasement of active compound in digestive system	Reduction in daily administrated medicine to one per day	The risk of abuse (crushable, can be dissolved and delivered intravenously or intranasally)	Moderate
Transdermal patches	Skin absorption (applied on the hip)	Flexible modulation of the duration of the pharmaceutical agent, Can be used by patients with gastrointestinal problems and dysphagia, Averts possible irritation of digestive system, Hepatoprotective properties, Possible abuse reduction	Skin irritation	Low
Serdexmethylphenidate	Oral administration, biotransformation to methylphenidate	No data	Minimal utility in the treatment of ADHD	Low

**Table 3:** Studies on ADHD and its impact on substance use disorders.

Administrated Drug	Participants	Form and Dose of Administrated Drug	Duration of Admission	Results	Limitations of Study	References
Methylphenidate	14 adults	Transdermal patches (1.1 mg/h—1st week; 1.6 mg/h—2nd week; 2.2 mg/h—rest of weeks)	8 weeks	Stimulant substances positive in 0/107 urine samples (1 self-report); Presence of non-stimulants (marijuana, opioids, and benzodiazepines) in 5/107 urine samples	Limited number of participants, may be not representative	[108]
Amphetamine salts	126 participants	60 mg or 80 mg extended-release AMPs	14 weeks of daily doses	60 subjects achieved cocaine abstinence	Not large sample of subjects	[130]
	2889 patients	Modafinil, methylphenidate, or AMPSs—varying between 38 trials	Systematic review, meta-analysis—varying between trials	Cocaine-negative urine samples: 281 (AMP treatment) vs. 216 (placebo usage)	Subjects without ADHD; included modafinil in paper, in results only included AMP	[132]
	28 patients	80 mg	12 week	AMP-administrated group—a significant decrease in cannabis use days over time compared to placebo	Small sample size	[134]
Lisdexamphetamine	36 adults	Orally: 50, 100 and 150 mg LDX; 40 mg dAMP and 200 mg diethylpropion; and placebo	6 study treatments (with different drugs) with 48 h interval	DRQS-liking score: 100 mg dose of LDX << 40 mg dose of d-AMP 150 mg of LDX ≈ AMP	Results subjective to patients’ feelings; No long-term research; no inclusion of pharmacokinetic measures	[119]
	12 males	Intravenous: 25 mg LDX, 10 mg d-AMP or placebo	3 study treatments with 48 h interval	On 5 five ARCI subscales LDX comparable to placebo	Enrolment of only males; Results subjective to patients’ feelings; No long-term research	[120]
	24 subjects	Orally: 100 mg LDX, 40 mg AMP or placebo	One dose	Plasma concertation of active ingredient similar between LDX and AMP	Use of only high does; No long-term research	[89]
	155 participants	Orally 250 mg LDX orally	12 weeks, plus 1-week induction and 2-week taper	OR of previous days of use of previous 28 days at week 13 between ones treated with LDX and placebo is 0.68	43% of subjects did not remain on medication; mostly based on self-report; limited consumer engagement	[136]
Not-stated stimulants	579 children born between 1994 and 1996	Not stated—cohort study	Data required at age of subject: 3 and 9 months, 2, 3, 6, 8, 10, 12, 14, and 16 years	Once subjects reached early adulthood number of patients misusing stimulants stopped increasing both ADHD patients and non-ADHD subjects	Administration of stimulants were only reported by parents (or self-reported once subject reached 18 years old) and are not backed by medical data	[129]
Stimulants	303 patients	Mostly immediate-release MPH, but also extended-release MPH and d-AMP	Follow-up on average 4.2 years after enrolment	Patients treated intensively at early age are more 0.28 more likely to develop SUD compared to naïve treatment	Risk of endogeneity; no distinction of different SUD	[135]
Atomoxitine	70 adolescents	Subjects’ weight < 70 kg: start at 0.5–0.75 mg/kg per day and increased by 25 mg per week until total dose 1.1–1.5 mg/kg. Participants’ weight > 70 kg: start at 50 mg per day and increased to 75 mg per day in the second week and 100 mg in the third week	12 weeks	No difference between atomoxetine and placebo group in terms of days of non-nicotine substance use	Not tested in adult population; only include non-nicotine substance use	[145]
Bupropion	14 adolescent boys	Start at 100 mg bupropion twice a day; Second week: 100 mg bupropion three times a day (if previous dose tolerated)	5 weeks	Two subjects with positive urine drug screen test	Not tested in adult population; only male as participants small sample size, lack of; controls or blinded assessments; assessments performed only before and after treatment	[154]
	13 adolescent outpatients	Start at 100 mg bupropion once-daily and then increased in time to 400 mg bupropion	6 months	13 adolescents: 0 or 1 prescribed drug misuse self-report	Not tested in adult population; possible observatory bias; small sample size; limited medical information	[155]

Abbreviations explained: LDX—Lisdexamphetmine; AMPs—amphetamine salts; MPH—methylphenidate; d-AMP—dextroamphetamine.

## Data Availability

No new data were created or analyzed in this study.

## Abbreviations

The following abbreviations are used in this manuscript:

ADHD	Attention-deficit/hyperactivity disorder
SUD	Substance Use Disorder
NICE	National Institute for Health and Care Excellence
CNS	Central Nervous System
CIUS	Compulsive Internet Use Scale
EEG	Electroencephalogram
MPH	Methylphenidate
LDX	Lisdexamphetamine
SDX	Serdexmethylphenidate
ATX	Atomoxetine
NRI	Norepinephrine reuptake inhibitors
fMRI	magnetic resonance imaging
